# Downregulation of CD147 by chitooligosaccharide inhibits MMP-2 expression and suppresses the metastatic potential of human gastric cancer

**DOI:** 10.3892/ol.2014.2115

**Published:** 2014-05-07

**Authors:** ZHIGUO LUO, XIAOXIA DONG, QING KE, QIWEN DUAN, LI SHEN

**Affiliations:** 1 Department of Clinical Oncology, Taihe Hospital, Hubei University of Medicine, Shiyan, Hubei 442000, P.R. China; 2 Department of Pharmacology, Hubei University of Medicine, Shiyan, Hubei 442000, P.R. China; 3 Department of Biochemistry, Hubei University of Medicine, Shiyan, Hubei 442000, P.R. China

**Keywords:** chitooligosaccharide, gastric cancer, metastasis, CD147, MMP-2

## Abstract

Metastasis is considered to be the major cause of mortality in patients with cancer, and gastric cancer is a highly metastatic cancer. In the present study, the anti-metastatic activity of chitooligosaccharide (COS) in human gastric cancer cells and its underlying mechanism were investigated. It was found that COS significantly inhibited SGC-7901 cell proliferation and metastasis in a dose-dependent manner, as observed by MTT, wound-healing and Transwell assays. Quantitative real-time polymerase chain reaction and western blot analysis indicated that COS could decrease the expression of cluster of differentiation 147 (CD147) and subsequently reduce matrix metalloproteinase-2 (MMP-2) expression. A clear dose-dependent inhibition of MMP-2 activity was also observed in SGC-7901 cells following treatment with COS in gelatin zymography experiments. Furthermore, overexpression of CD147 (when transfected with pEGFP-C1 plasmid) in SGC-7901 cells partially protected against COS-induced inhibition of MMP-2. The results of the present study demonstrated the potential of COS in suppressing gastric cancer metastasis, and that the CD147/MMP-2 pathway may be involved as the key mechanism of its anti-metastatic effect.

## Introduction

Being a worldwide malignant liver tumor, gastric cancer ranks the fourth in frequency among common human solid tumors and the second leading cause of cancer-related mortality (1). In China, gastric cancer incidence ranks second only to lung cancer and accounts for approximately half of the global incidence. In spite of the improvement in surgical and multimodal therapy, the prognosis of advanced gastric cancer remains poor due to the recurrence, invasion and metastasis, with a 5-year survival rate of <30%. An improved knowledge of the mechanisms underlying tumor metastasis is warranted to discover novel paradigms for the diagnosis and treatment of gastric cancer. Cancer metastasis is a highly complex process that occurs through multiple steps, which include cell invasion, cell migration, intravasation, transport through the circulatory system, arrest at a secondary site, extravasation and growth in a secondary organ (2). Numerous metastasis-promoting genes are embedded in the primary tumors and the ability to metastasize may be an inherent quality of the tumor from the start. Additionally, multiple genes have been reported to be involved in the metastasis of gastric cancer.

Matrix metalloproteinases (MMPs) have been regarded as major critical molecules that assist tumor cells during metastasis. MMPs, also designated matrixins, are proteinases that participate in extracellular matrix (ECM) degradation. They selectively cleave polypeptide bonds in ECM and remodel structural proteins that are essential for the maintenance of connective tissue integrity, such as collagens, aggrecan, fibronectin, proteoglycan and laminin. To date, 24 different vertebrate MMPs have been identified, 23 of which are found in humans (3). Among the numerous MMPs that have been identified, MMP-2 encodes an enzyme which degrades type IV collagen, the major structural component of basement membranes. MMP-2 overexpression was found in a large proportion (94%) of the gastric cancer tissues compared with the matched non-cancerous tissues (4). An increase in MMP-2 levels and the presence of the active type of MMP-2 were closely associated with the ability of invasion and metastasis of gastric cancer (5).

Cluster of differentiation 147 (CD147), a 55-kDa transmembrane glycoprotein, is located on the surface of human tumor cells and normal keratinocytes (6). CD147 has been implicated in tumor invasion and its elevated levels in cancer tissues have been correlated with tumor progression in numerous malignant tumor models (7). For example, overexpression of CD147 has been demonstrated to enhance the metastatic potential in human hepatoma cells (8). The silencing of CD147 expression in a murine B16 melanoma model resulted in a reduced capability of the tumor cells to metastasize to the draining lymph nodes (9). Gastric cancer tissue with higher CD147 expression also displayed an increased ability to invade into lymphatic or venous vessels, or through the gastric wall (10). Downregulation of CD147 by RNAi led to decreased cell proliferation, and invasive potential of SGC7901 cells (11). Therefore, control of CD147 expression has considerable significance for regulation of the metastatic capacity of gastric cancer.

Chitooligosaccharide (COS) is a natural alkaline polysaccharose, an oligosaccharide formed by 2–10 amino-glucoses through 1,4-glucosidic bond connection (12). The water-soluble COS possess various biological activities, such as antitumor activity, antimicrobial activity and antimutagenic activity (13). However, to date, no study has reported the anti-metastatic effect of COS and its underlying mechanism in human gastric cancer. In the present study, several different gastric cancer cell lines were tested first for their sensitivity to growth inhibition by COS. It was found that SGC-7901 was the most sensitive cell line among the tested cancer cell lines. Then, the molecular mechanisms by which COS inhibited SGC-7901 cell proliferation and metastasis were investigated. Results presented here suggested that CD147/MMP-2 pathway played an important role in the treatment of COS. We propose that COS has the potential to be a novel chemotherapeutic agent for gastric cancer.

## Materials and methods

### Materials

COS (1 kDa<MW<3 kDa) was obtained from the Dalian Institute of Chemical Physics, the Chinese Academy of Sciences (Dalian, China). 3-(4,5-Dimethylthiazol-2-yl)-2,5-diphenyltetrazolium bromide (MTT) was purchased from Sigma-Aldrich (St. Louis, MO, USA) and Transwell chambers were purchased from Corning Inc. (Corning, NY, USA). All reagents used for cell culture were obtained from Gibco BRL (Grand Island, NY, USA). Monoclonal goat anti-human CD147, monoclonal rabbit anti-human MMP-2 and monoclonal mouse anti-human GADPH antibodies were purchased from Santa Cruz Biotechnology, Inc. (Santa Cruz, CA, USA).

### Cell culture

The SGC-7901 human gastric cancer cell line was obtained from the Type Culture Collection of Chinese Academy of Sciences (Shanghai, China). AGS and NCI-N87 human gastric cancer cells were purchased from American Type Culture Collection (Rockville, MD, USA). All the cells were grown in RPMI-1640 (Invitrogen Life Technologies, Carlsbad, CA, USA) supplemented with 10% fetal calf serum at 37°C in a 5% CO_2_ incubator.

### Plasmid construction and transfection

For CD147 overexpression, CD147 was constructed into a pEGFP-C1 eukaryotic expression vector (Clontech, Heidelberg, Germany). Transfection was carried out using Lipofectamine 2000 (Invitrogen Life Technologies), according to the manufacturer’s instructions. The cells were selected in the presence of 500 mg/l G418 (Invitrogen Life Technologies). Stable clones were selected for at least 4 weeks before single colonies were picked and analyzed for CD147 expression by western blotting. The empty vector pEGFP-C1 was also transfected into SGC7901 cells, which served as the control group.

### MTT assay

Cells were seeded into 96-well plates at a density of 1×10^5^ cells/100 μl/well. After 24 h growth, cells were treated with different concentrations of COS (50, 100, 250, 500 and 1,000 μg/ml) for 24, 48 and 72 h. Following the addition of 100 μl MTT (0.5 mg/ml; Sigma-Aldrich), cells were incubated at 37°C for 1 h. The formazan deposits that formed were solubilized in DMSO, and the absorbance of each well was measured at 570 nm in an EMax Precision microplate reader (Molecular Devices Instruments, Sunnyvale, CA, USA).

### Wound-healing assay

Cells were cultured in a 24-well plate with 100% confluency. A micro-pipette tip was used to scratch a line in the cell monolayer. The medium was removed and the monolayer was washed with warm phosphate-buffered saline three times. Then growth medium containing different concentrations of COS was added to each well. Following incubation for 48 h, cell migration was observed and photographs were taken under a light microscope (Olympus BX41; Olympus Corporation, Tokyo, Japan).

### Transwell assay

Cell migration through Matrigel-coated filters was measured by using Transwell chambers (Costar Corporation, Tewksbury, MA, USA) with 8-μm-pore polycarbonate filters coated with Matrigel matrix. SGC-7901 cells were seeded in the upper compartment of each invasion chamber and incubated in the presence of COS for 48 h. The lower well was filled to the top (500 μl) with RPMI-1640 containing 10% fetal calf serum (Hangzhou Sijiqing Bioengineering Material Co., Ltd., Hangzhou, China) as a chemoattractant. Subsequently, non-migrating cells on the upper surface of the membrane were removed by gently scrubbing with a cotton swab, and the invading cells on the lower surface were fixed with 100% methanol and stained with crystal violet (0.1%; Beyotime Institute of Biotechnology, Nantong, China). The number of cells was counted under a light microscope (Olympus BX41; Olympus Corporation) at a magnification of ×100. Images were captured and subjected to computer-assisted image analysis using a computer coupled to the Olympus BX41 light microscope (Olympus Corporation) using AnalySis software (Olympus Corporation).

### Quantitative real-time polymerase chain reaction (qPCR)

Total RNA of cells with or without COS intervention was extracted using TRIzol reagent (Invitrogen Life Technologies). Subsequently, 2 μg of total RNA was reverse transcribed in a total volume of 20 μl containing 200 units of SuperScript II RNase H- Reverse Transcriptase (Invitrogen Life Technologies), 50 mM Tris-HCl (pH 8.3), 75 mM KCl, 3 mM MgCl_2_, 10 mM dithiothreitol, 500 μM dNTPs (each), 500 ng oligo (dT) 23 primer and 40 units of RNaseOUT, at 42°C for 50 min. This was then followed by inactivating at 70°C for 15 min. qPCR was conducted by LightCycler 480 SYBR Green I Master (Roche Diagnostics, Indianapolis, IN, USA) with 1 mM primers. The expression of GADPH was used as endogenous control for the normalization of gene expression. All reactions were performed in duplicate. The forward and reverse primers were 5′-CCCCAAAACGGACAAAGAC-3′ and 5′-CTTCAGCACAAACAGGTTGC-3′, respectively, for human MMP-2; 5′-CCATGCTGGTCTGCAAGTCAG-3′ and 5′-CCGTTCATGAGGGCCTTGTC-3′, respectively, for human CD147; and 5′-CCAACCGCGAGAAGATGA-3′ and 5′-CCAGAGGCGTACAGGGATAG-3′, respectively, for human GADPH.

### Western blot analysis

Whole cell lysates from cultured cells were harvested with cell lysis buffer. Equal amounts of protein (20 μg) were separated by SDS-PAGE produced in-house and transferred to a polyvinylidene difluoride membrane (Millipore, Bedford, MA, USA). The membrane was blocked with 5% skimmed milk in Tris-buffered saline and then incubated with primary antibodies for 1 h at room temperature. Horseradish peroxidase-conjugated monoclonal goat anti-rabbit, goat anti-mouse and donkey anti-goat secondary antibodies and an enhanced chemiluminescence kit (all Beyotime Institute of Biotechnology) were used for detection.

### Gelatin zymography

Gelatin zymography was performed to determine the activity of MMP-2. Briefly, protein in medium was then separated in 10% SDS-PAGE gel containing 1 mg/ml gelatin (Sigma-Aldrich) at 4°C. After running, the gel was incubated in 2.5% Triton X-100 (Beyotime Institute of Biotechnology) in deionized water for renaturing with gentle agitation for 30 min at room temperature. Subsequently, the gel was incubated in developing buffer (50 mM Tris-HCl, 0.2 M NaCl, 5 mM CaCl_2_ and 0.02% Brij35) overnight with gentle shaking. The gel was stained with Coomassie blue R-250 (Beyotime Institute of Biotechnology) for 30 min and then washed. Gelatinolytic bands were observed as clear zones against the blue background and the intensity of the bands was evaluated using ImageMaster software (Amersham Pharmacia Bioscience, NJ, USA).

### Statistical analyses

Data are expressed as the mean values ± standard deviation from at least three experiments. Statistical comparisons were based on Student’s t-test or analysis of variance. P<0.05 was considered to indicate a statistically significant difference.

## Results

### Effects of COS on cell proliferation in AGS, SGC-7901 and NCI-N87 cells

**Figure 1:**
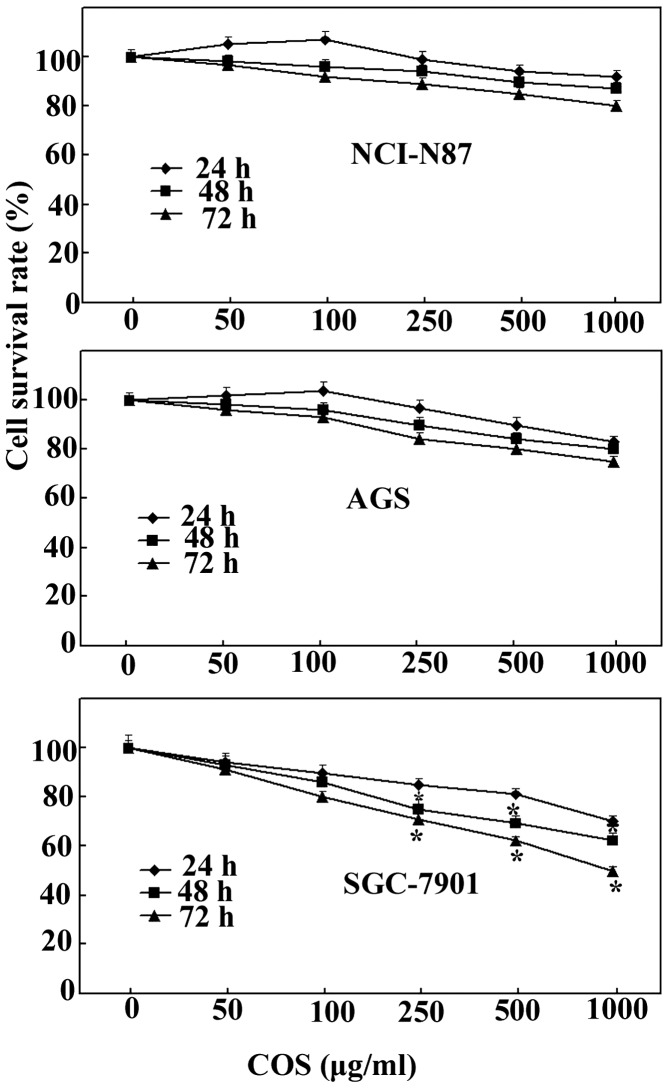
Effects of different concentrations of COS on cell viability in cultured NCI-N87, AGS and SGC-7901 cells. The proliferation of three cell lines were quantified by the cell survival rate after 24, 48 and 72 h of incubation with concentrations of COS ranging from 50 to 1000 μg/ml, assessed by 3-(4,5-Dimethylthiazol-2-yl)-2,5-diphenyltetrazolium bromide assay. Data are presented as means of values ± SD from three independent experiments. ^*^P<0.05 vs. 0 μg/ml COS treatment group. COS, chitooligosaccharide.

To investigate the antiproliferative effect of COS on various human gastric cancer cells, an MTT assay was carried out following treatment with different concentrations of COS ranging from 50 to 1000 μg/ml. As shown in Fig. 1, COS treatment marginally inhibited cell growth in AGS and NCI-N87 cells, but significantly inhibited cell growth in SGC-7901 cells. COS inhibition of SGC-7901 cell growth was dose and time-dependent; however, the effect became significant only after ≥48 h of treatment. Notably, COS concentrations >250 μg/ml exhibited a marked inhibitory effect on cell proliferation compared with low doses of COS (50 and 100 μg/ml). Therefore, SGC-7901 cells were treated with COS at concentrations of 250, 500 and 1,000 μg/ml for 48 h in the following experiments.

### Effects of COS on cell migration and invasion

**Figure 2:**
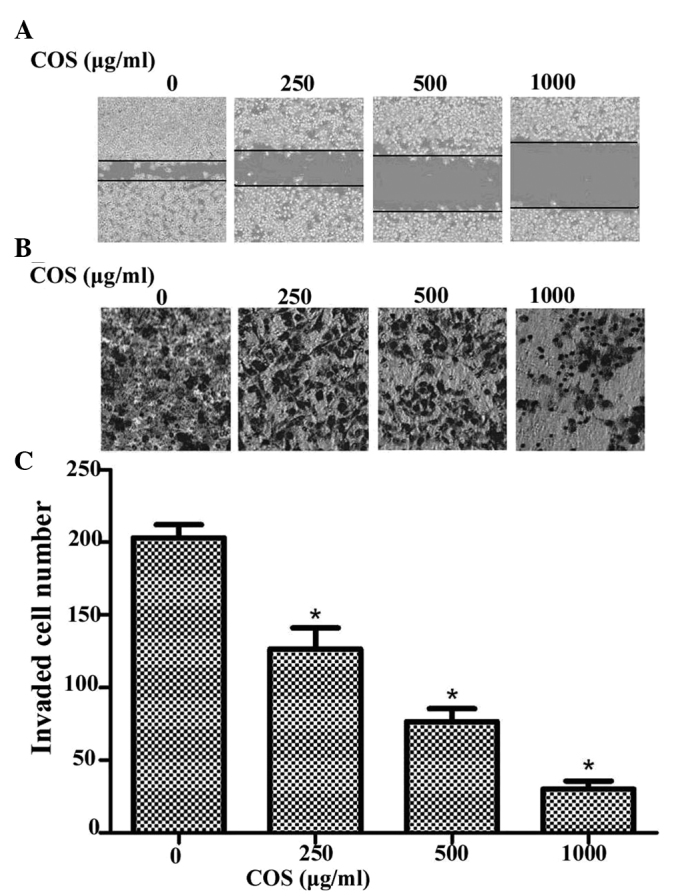
COS inhibits SGC-7901 cell migration and invasion. (A) COS markedly reduces SGC-7901 cell migration in dose-dependent manner. (B) Representative photographs of invasive SGC-7901 cells on the membrane (magnification, ×100). (C) Average number of invasive SGC-7901 cells. (^*^P<0.05, compared with the 0 μg/ml COS treatment group). COS, chitooligosaccharide.

In order to further assess the influence of COS on SGC-7901 cells, cell migration and invasion assays were employed to determine these two key factors of malignant progression and metastasis. The wound healing assay showed that treatment of SGC-7901 cells with increasing concentrations of COS after 48 h led to a concentration-dependent decrease in wound-healing cell migration (Fig. 2A). COS also caused a dose-dependent decrease in the invasion of SGC-7901 cells through the Matrigel chamber after 48 h (Fig. 2B). The number of invasive cells was significantly decreased following COS treatment in a dose-dependent manner (P<0.05, compared with the 0 μg/ml COS treatment group) (Fig. 2C). These results indicate that COS is a potential inhibitor of metastasis of gastric cancer cells.

### Effects of COS on CD147 and MMP-2 expression

**Figure 3:**
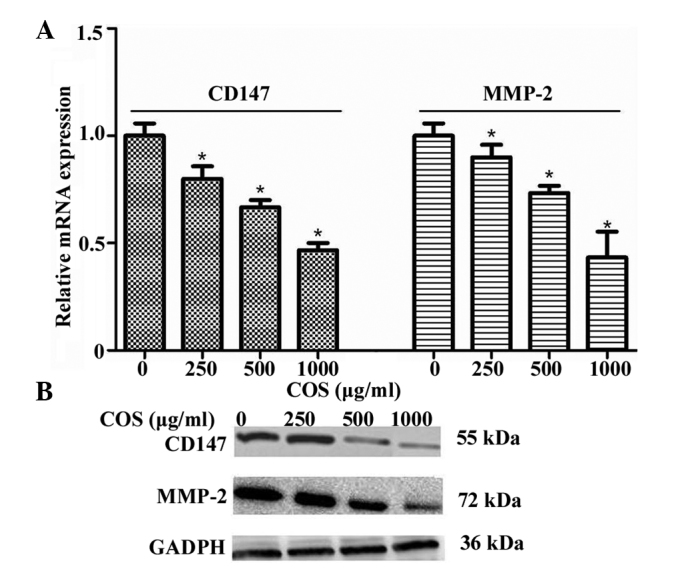
COS represses the mRNA and protein expression of CD147 and MMP-2 in dose-dependent manner. SGC-7901 cells were treated for 48 h with COS and analyzed for the indicated (A) mRNA by quantitative real-time polymerase chain reaction and (B) protein by western blotting. The results represent the mean ±SD of experiments performed in triplicate. (^*^P<0.05, compared with the 0 μg/ml COS treatment group). COS, chitooligosaccharide; CD147, cluster of differentiation 147; MMP-2, matrix metalloproteinase-2.

Based on the abovementioned results, further studies were carried out to examine the inhibitory effect of COS on CD147 mRNA and protein expression in SGC-7901 cells. Total RNA of cells was isolated and qPCR was performed as described in Materials and methods. As shown in Fig. 3A, the levels of CD147 mRNA were significantly and dose-dependently suppressed by COS. Pretreatment of SGC-7901 cells with COS also decreased the protein expression of CD147 in a concentration-dependent manner, which was detected by western blot analysis (Fig. 3B). Furthermore, the change of MMP-2 at the levels of mRNA and protein expression displayed a similar trend to that of CD147 (Fig. 3A and B). In particular, 1,000 μg/ml COS exhibited the greatest inhibitory effect on CD147 and MMP-2 expression.

### Effects of COS on MMP-2 activity

**Figure 4:**
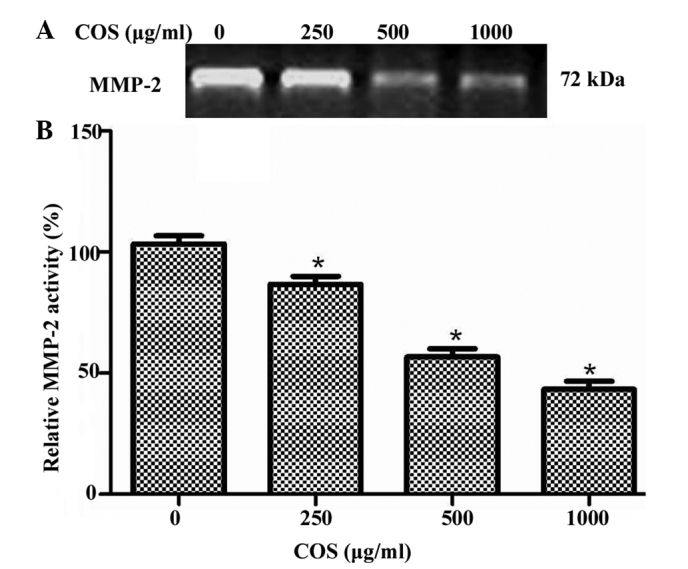
COS treatment downregulates the activity of MMP-2 in SGC-7901 cells. (A) SGC-7901 cells were treated with various concentrations of COS for 48 h, and the activity of MMP-2 was evaluated by gelatin zymography. (B) Areas and relative intensities of gelatin-digested bands by MMP-2 were quantified by densitometry and expressed as relative activity. The results represent mean ± SD of experiments performed in triplicate. (^*^P<0.05, compared with the 0 μg/ml COS treatment group). COS, chitooligosaccharide; MMP-2, matrix metalloproteinase-2.

Increased MMP-2 activity is considered to be important for the increased capability of gastric cancer cells to traverse the membrane and invade and metastasize to distant sites (14). Therefore, the present study analyzed the effect of COS on the activity of MMP-2 cells. SGC-7901 cells were treated with different concentrations of COS (0, 250, 500 and 1,000 μg/ml), and the activity level of MMP-2 was determined by gelatin zymography assay. As shown in Fig. 4A, the inhibitory effect on enzymatic activity of MMP-2 was increased with increasing concentrations of COS. Quantification analysis indicated that the MMP-2 activity reduced by 17.6, 37.5 and 52.3% when cells were treated with 250, 500 and 1,000 μg/ml of COS, respectively (Fig. 4B). Notably, 1,000 μg/ml COS exhibited the greatest inhibitory effect on MMP-2 activity.

### CD147 overexpression can attenuate the effect of COS on MMP-2 inhibition

**Figure 5:**
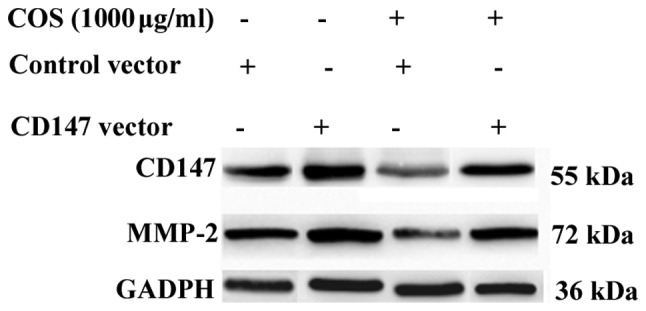
CD147 overexpression could attenuate the effect of COS on MMP-2 inhibition. SGC-7901 cells were treated for 48 h with 1000 μg/ml COS and analyzed for the indicated protein by western blotting. GADPH was used as the internal control. All assays were performed in triplicate. CD147, cluster of differentiation 147; COS, chitooligosaccharide; MMP-2, matrix metalloproteinase-2.

To further elucidate the mechanism by which COS mediates MMP-2 inhibition through CD147, exogenous CD147 was introduced into SGC-7901 cells. As shown in Fig. 5, SGC-7901 cells were treated for 48 h with COS at a concentration of 1000 μg/ml and then analyzed for the indicated protein by western blotting. CD147 overexpression activated the expression of MMP-2, and COS-induced MMP-2 inhibition was reduced by CD147 overexpression. The results indicate that the CD147/MMP-2 pathway is likely to be an important target of COS in SGC-7901 cells.

## Discussion

Chitosan, which is a linear polysaccharide composed of randomly distributed β-(1–4)-linked D-glucosamine (deacetylated unit) and N-acetyl-D-glucosamine (acetylated unit), has been widely used in the food, pharmaceutical, agricultural and environmental industries. In recent years, chitosan has attracted interest when converted to COS, as COS is not only water-soluble and of low molecular weight but has also been demonstrated to exhibit various biological functions (13,15,16). The majority of these studies suggested that COS exhibited antimetastatic activity *in vivo* and *in vitro* (15,16). However, the underlying mechanisms and the direct influence of COS on gastric cancer cells have not been fully tested in detail. In the present study, we demonstrated that COS treatment marginally inhibited cell growth in AGS and NCI-N87 cells, but significantly inhibited cell growth in SGC-7901 cells. A similar result has been reported by Karagozlu *et al* (17); however, the detailed mechanisms remain unclear. In the current study, it was found that SGC-7901 was the most sensitive cell line among the tested cancer cell lines. The wound-healing and Transwell assays further confirmed that COS could inhibit the metastatic process of SGC-7901 cells.

Expression of various MMPs has been found to be upregulated in virtually every type of human cancer and correlates with advanced stage, invasive and metastatic properties and, in general, poor prognosis. Further upregulation of MMP expression, in particular the gelatinases, which can degrade basement membrane components, allows the tumor cells to invade into the adjacent stroma and to break down the basement membranes associated with capillaries and lymphatic vessels, allowing the tumor cells to enter the circulation (18). Therefore, compounds that have the potential to regulate MMPs are considered to be attractive targets for therapeutic intervention. Numerous studies have confirmed that the expression and activity of MMPs could be mediated by COS. For example, Kim and Kim found that COS suppressed the protein expression of MMP-2, and this inhibition was caused by a decrease in the gene expression and transcriptional activity of MMP-2 (19). MMP-9 inhibition in the presence of COS has been clearly observed in HT1080 cells among tested molecular mass fractions (20). Furthermore, the inhibitory effect of MMP-9 observed in HUVEC cells (21) confirms that COS exerts its effect regardless of cell type. In the present study, the expression of MMP-2 mRNA and protein, as measured by qPCR and western blotting, was downregulated by COS in SGC-7901 cells at concentrations of 250, 500 and 1,000 μg/ml (P<0.05). It was also observed that COS caused a decline in the enzymatic activity of MMP-2. These data demonstrate that COS can significantly repress the invasion and migration ability of gastric cancer cells in a dose-dependent manner, and this repression strongly correlates with the inhibition of MMP-2.

As a tumor-associated antigen, CD147 forms homo-oligomers in both heterotypic and homotypic cell-cell interactions to induce production of MMPs. The functional importance of CD147 has been demonstrated to be associated with its ability to stimulate MMP expression. CD147 can induce the production of MMP-1, MMP-2, MMP-3, MMP-9, MMP-14, and MMP-15 (22). Supporting its key role in the processes of tumorigenesis and metastasis, CD147 has been reported to be one of the most constantly upregulated mRNAs in metastatic cells (23). Downregulation of CD147 expression by RNA interference has been demonstrated to inhibit MMP-2 expression and suppress cell proliferation, invasion and tumorigenicity *in vitro* and *in vivo* (24). In SGC-7901 gastric cancer cells, silencing the CD147 gene was found to significantly decrease the proliferation and invasion of cells, and downregulate the activity of MMP-2 (25). These studies support a model in which CD147 in tumor cells stimulates MMP-2 production, thereby leading to ECM degradation and increased tumor growth and metastasis. To better understand how the COS regulated MMP-2, the present study analyzed the change in CD147 expression levels following treatment with different concentrations of COS ranging from 250 to 1,000 μg/ml. The results revealed that the levels of CD147 were significantly and dose-dependently suppressed by COS. The change in MMP-2 expression levels displayed a similar trend to that of CD147. These results therefore suggested that control of CD147 expression by COS is involves in determining MMP-2 expression in cancer metastasis.

As the regulation of gene expression of MMP-2 is controlled by CD147, the current study next investigated whether overexpression of CD147 could attenuate the effect of COS on MMP-2 inhibition. It was found that this effect could be effectively attenuated by CD147 overexpression, suggesting that the downregulating role of COS in MMP2 expression may be through the upregulation of CD147. Further characterization of the effect of COS on gastric cancer invasion and metastasis may lead to the identification of new diagnostic markers and therapeutic targets. We propose that these results may apply to a number of additional cancer types other than gastric cancer, as CD147 and MMP-2 are frequently upregulated in numerous other cancer types as well.

In conclusion, to the best of our knowledge, the present study is the first to demonstrate that COS exhibits an anti-metastatic effect in human gastric cancer. The effects of COS on cell invasion and migration ability may be achieved through the inhibition of MMP-2 expression by decreasing CD147. Overexpression of CD147 partially protected against COS-mediated inhibition of MMP-2. Overall, our findings have demonstrated the potential of COS in suppressing gastric cancer metastasis and that the CD147/MMP-2 pathway may be involved as the key mechanism of its anti-metastatic effect.
